# Integrated Management of European Cherry Fruit Fly *Rhagoletis cerasi* (L.): Situation in Switzerland and Europe

**DOI:** 10.3390/insects3040956

**Published:** 2012-10-16

**Authors:** Claudia Daniel, Jürg Grunder

**Affiliations:** 1 Research Institute of Organic Agriculture (FiBL), Ackerstrasse 21, Postfach 219, CH-5070 Frick, Switzerland; 2 Zurich University of Applied Sciences (ZHAW), Department of Natural Resources Sciences, Grueental, P.O. Box 335, CH-8820 Waedenswil, Switzerland; E-Mail: grng@zhaw.ch

**Keywords:** *Rhagoletis cerasi*, Diptera, Tephritidae, management, IPM, organic, biology, antagonists, mortality

## Abstract

The European cherry fruit fly, *Rhagoletis cerasi* (L.) (Diptera: Tephritidae), is a highly destructive pest. The low tolerance for damaged fruit requires preventive insecticide treatments for a marketable crop. The phase-out of old insecticides threatens cherry production throughout the European Union (EU). Consequently, new management techniques and tools are needed. With the increasing number of dwarf tree orchards covered against rain to avoid fruit splitting, crop netting has become a viable, cost-effective method of cherry fruit fly control. Recently, a biocontrol method using the entomopathogenic fungus *Beauveria bassiana* has been developed for organic agriculture. However, for most situations, there is still a lack of efficient and environmentally sound insecticides to control this pest. This review summarizes the literature from over one hundred years of research on *R. cerasi* with focus on the biology and history of cherry fruit fly control as well as on antagonists and potential biocontrol organisms. We will present the situation of cherry fruit fly regulation in different European countries, give recommendations for cherry fruit fly control, show gaps in knowledge and identify future research opportunities.

## 1. Introduction

The European cherry fruit fly, *Rhagoletis cerasi* (L.) (Diptera: Tephritidae) is the most important pest of sweet cherries in Europe. Without insecticide treatment, up to 100% of fruits can be infested [1]. *R. cerasi* poses a challenge to cherry growers because the tolerance level of the market for damaged fruit is relatively low, with a maximum of 2% of infested fruits. Because fruit fly infested fruit cannot be sorted out, the whole lot is rejected if tolerance levels are exceeded. The disqualification of table cherries to distillery quality considerably reduces the market price, which causes serious financial losses. The low tolerance level is the principal reason for preventive insecticide treatments. The regulatory phase-out of “old” insecticides now threatens cherry production throughout the European Union (EU). The currently used insecticide dimethoate in particular is being challenged due to problems of ecotoxicity and residues. Yellow sticky traps are currently used as an alternative in organic cherry production. However, this strategy is labor-intensive and often does not provide sufficient control [2]. This review will explore the literature of research on *R. cerasi* conducted between 1891 and 2012. In it, we summarize the biology and history of cherry fruit fly control as well as research on antagonists and potential biocontrol organisms. Finally, we will present current practices to control cherry fruit flies in different European countries, recommend strategic practices to reduce cherry fruit fly populations, identify knowledge gaps, and suggest topics suitable for future research.

## 2. Taxonomy, Distribution and Host Plants of *R. cerasi*

**Figure 1:**
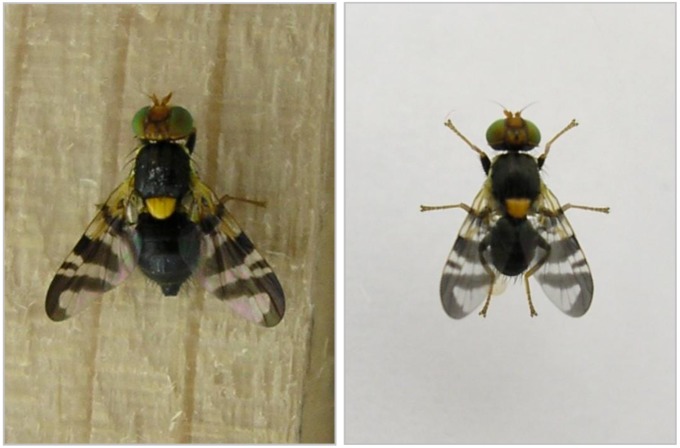
Adult *R. cerasi*: female (**left**) and male (**right**) with its bright black thorax, yellow scutellum and characteristic wing pattern and a size of 4mm (males) to 5mm (females).

The European cherry fruit fly (Figure 1) belongs to the family of Tephritidae, which has a worldwide distribution of about 4,000 described species in about 500 genera [3]. The genus *Rhagoletis* Loew includes about 65 known species [4]. Most species are oligophagous, attacking only a few closely related host plants. In addition to *R. cerasi*, the American cherry fruit fly species *R. cingulata*, *R. indifferens* and *R. fausta*, as well as the apple maggot *R. pomonella*, the blueberry maggot *R. mendax*, and the walnut infesting species *R. completa* and *R. suavis* are pest insects of economic importance [5]. Host plants of *R. cerasi* include various different *Prunus *sp. (Rosaceae; *P. cerasus*, *P. avium*, *P. serotina*, *P. mahaleb*) [6,7] as well as *Lonicera *sp. (Caprifoliaceae; *L. xylosteum* and *L. tatarica*) [4,8,9,10,11,12].

*R. cerasi* is distributed throughout Europe and temperate regions of Asia [4,13]. Boller *et al*. [14] assumed that there are two races, which were referred to as the northern and southern race. The southern race is found in Italy, Switzerland and Southern Germany, whereas the northern race ranges from the Atlantic Ocean to the Black Sea [4]. However, Riegler and Stauffer [15] showed that the unidirectional cytoplasmatic incompatibility is caused by maternally inherited *Wolbachia* infections. As a consequence, southern females and northern males are interfertile, but crosses between southern males and northern females are sterile [14,15,16,17,18,19,20].

Recently, the American cherry fruit fly species *Rhagoletis cingulata*, which is closely related to the European cherry fruit fly *R. cerasi*, was introduced to Europe [21,22,23,24,25]. One individual was first observed in Switzerland (canton Ticino) in the 1980s. From 1991 to 1993, there were repeated captures of American cherry fruit flies in the south of the canton Ticino [26]. Until now, no stable populations are known in Switzerland [27]. However, this may be due to insufficient monitoring intensity. A close monitoring in the Rhine Valley (Rheinhessen, Germany) from 2002 to 2004 revealed that the American cherry fruit fly was widespread and established in many orchards [28,29,30]. In 2007 it was first detected in Austria [25]. The American species has a similar biology to the European species. The only differences are that the peak flight activity of the American species occurs two weeks later than the peak flight activity of *R. cerasi*, eggs are deposited in yellow fruit, and sour cherries are also heavily attacked. 

## 3. Life History *R. cerasi*

Life history characteristics of *R. cerasi*, like those of other oligophagous Tephritid species, are best suited for exploiting resources that are predictable in time and space, but are only available during a short period of the year. A close adaptation of their biology to the fruiting pattern of the host and precision in seasonal synchronization are more important than high reproductive potential and high mobility [31]. Hibernation occurs in the soil in the immediate vicinity of the hosts. Thus there is no need for dispersal flights. Adult emergence and life span are closely correlated with host plant phenology [5]. Pupal carryover for two or more winters is used for “spreading the risk” of failure of the host plants to fruit in a particular year [31,32]. There is only one generation each year and a long obligatory winter diapause [33]. Fecundity is considered to be lower than in the polyvoltine Tephritid species [5]. Relatively unspecific visual and odor stimuli are used to identify oviposition sites. Competition in the larval stages (contest type) is largely avoided by oviposition of only a single egg in each fruit and by the application of a host marking pheromone after oviposition, which ensures an adjustment of larval density to the carrying capacity of the host and maximizes dispersion over available food resources [34]. The mating system of these species is usually resource-based: The males control the oviposition substrates, and mating is often initiated by forced copulation without elaborate courtship behavior [35].

### 3.1. Aspects of R. cerasi Biology Relevant for Its Management

**Emergence of adult flies and pre-oviposition period:** Pupal development and adult emergence is influenced by soil temperature in spring [6,7,36,37], by temperature conditions during winter diapause [38,39,40] as well as by the host plants from which the pupae originated [41,42,43,44] and geographic provenance [45,46]. In Switzerland, Austria and Southern Germany, the first flies usually appear in the orchards between mid-May and mid-June [47]. The earliest attempts to develop a forecasting model for the eclosion time of flies were made in the 1930s [48,49,50]. This model was revised and improved in the 1960s [7,51] and 1970s [38,45]. Before oviposition, the adults go through a temperature-dependent maturation period of six to 13 days [7,47,52,53,54,55,56] during which they need to feed on carbohydrates, proteins and water in order for the gonads to mature. Nutrients are obtained from bird feces, honeydew, extrafloral nectaries, and bacterial colonies on leaf and fruit surfaces [49,57,58,59,60,61]. In addition to the temperature and nutritional status of the females, the maturity stage of the cherries can also affect the beginning of oviposition [53]. The life span of flies under field conditions is difficult to estimate and may range between four to seven weeks [47,49,53,59], which leads to a total flight period of seven to 11 weeks [47,48,62].

**Figure 2:**
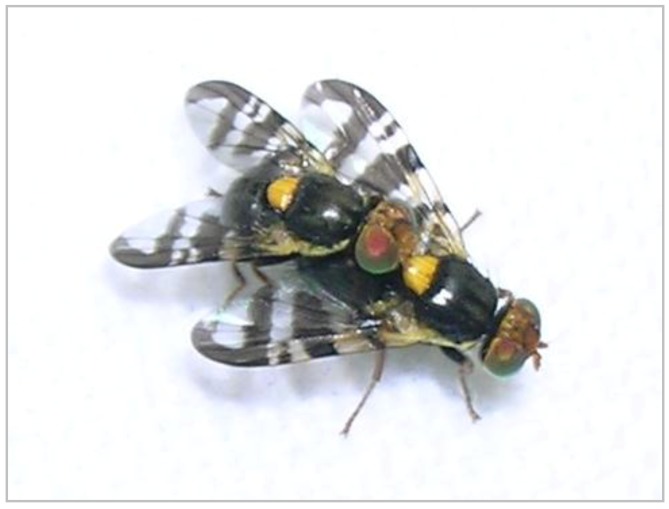
Mating of *R. cerasi*.

**Mating:** Mating (Figure 2) occurs on sunny days with temperatures above 15 °C [49,63]. Host fruit on sunny parts of the trees is used as a mating site. Mating is initiated when a female in search of an oviposition site lands on a fruit occupied by a male [63]. Thus, fly behavior plays a major role in locating mating partners: Due to their preference for host fruits in full sun, the flies aggregate in certain parts of the trees. In these circumstances, an elaborate long-range pheromone might be of minor importance [64]. Nevertheless, it was shown that the males produce a highly species-specific pheromone, which attracts females [63,64,65,66,67,68]. However, contrary to the pheromones of many Lepidoptera, this pheromone seems not to have a long-range attraction [64,66]. It was even hypothesized that the pheromone might function primarily as an aphrodisiac [5]. One to three copulations during a female’s life span are considered to be necessary to maintain high egg fertility [49].

**Dispersal and flight ability:** With the relative stability of the system, *i.e.*, pests that overwinter beneath perennial hosts, there appears to be little impetus for adults to move long distances. Dispersal flights occur only in situations in which flies are deprived of suitable fruits for oviposition: Such as when cherries are destroyed by frost or early harvest or when all fruits are already marked with the host-marking pheromone [69]. Driven by high oviposition pressure, the females leave their original tree [36], and the males follow a little later [49,69]. The flies move from tree to tree until they find a suitable host [55]. Maximum distances of dispersal flights are difficult to evaluate experimentally and might range between 100 and 500 m [7,55,70], in exceptional cases as far as 3 km [71]. Flight studies in the laboratory have shown that flies are capable of flying several kilometers in 24 h if no landing platforms are available [72]. However, within orchards, 95% of the flies move only to neighboring trees of later ripening varieties [7,73], and from there on to *Lonicera *sp. bushes [69].

**Orientation during dispersal flights:** Orientation during dispersal flight is mainly based on visual stimuli. Foliage color, tree shape and tree size play a role in eliciting the arrival of flies. *R. cerasi* is known to be highly responsive to visual stimuli [74], especially to yellow surfaces [70,75,76,77,78]. Prokopy [79] suggested that large yellow surfaces represent a super-normal foliage-type stimulus that elicits food-seeking behavior in *R. cerasi*. In addition to flat yellow surfaces, Prokopy [79] showed that *Rhagoletis* flies also react to red or dark colored spheres of approximately the same size as the host fruit [77,78]. Attraction of fruit flies to spherical objects is believed to represent a response to mating and oviposition site stimuli. However, none of these cues are host-specific. Boller [70] believes that the flies are not able to distinguish between host and non-host trees at greater distances, whereas Katsoyannos *et al*. [69] believes that females can identify trees with fruits at the right ripening stage from a certain distance. However, once the flies arrive at a host tree, they might be able to identify host-specific leaf stimuli with their tarsal contact chemoreceptors [80].

**Oviposition:** Oviposition occurs around noon and during the early afternoon [81] on sunny days when temperatures rise above 16 °C [44,47,49,82]. Weather conditions during the oviposition period are considered to be crucial for the regulation of population densities: The high oviposition activity during long-lasting periods of fine weather can lead to extreme outbreaks of this pest [50]. Both olfactory and visual cues are involved in the choice of suitable fruits for oviposition. However, the visual component appears to dominate. Females recognize the fruit by visual cues based on shape (spherical or hemispherical), size (2.5 to 10.3 mm diameter) and contrast-color against the background (dark shape in front of lighter background) [5,74,83,84]. Once a suitable fruit has been located, the female explores the surface structure (smoothness, softness and shape) by walking in circles on the surface and decides whether or not to oviposit [83,85]. During this exploration, the condition and the chemistry of a fruit might influence oviposition behavior. Cherries at the stage of color change from green to yellow, with a hardened cherry pit, and pulp at least 5 mm thick are preferred for oviposition [86]. The female pierces the fruit with its ovipositor and inserts a single egg just below the skin [87]. After oviposition the females deposit a water-soluble host-marking pheromone by dragging the ovipositor around the fruit surface [6,63,88]. This pheromone prevents further ovipositions into the same fruit [89,90,91]. Under field conditions with high infestation levels, however, multilarval infestations are frequently observed, which suggest multiple ovipositions into the same fruit [1,92,93]. Fecundity seems to depend mainly on the life span of females. Under field conditions, fecundity is thought to range from 30 eggs to as many as 200 eggs per female [7,47,48,49,56,82].

**Figure 3:**
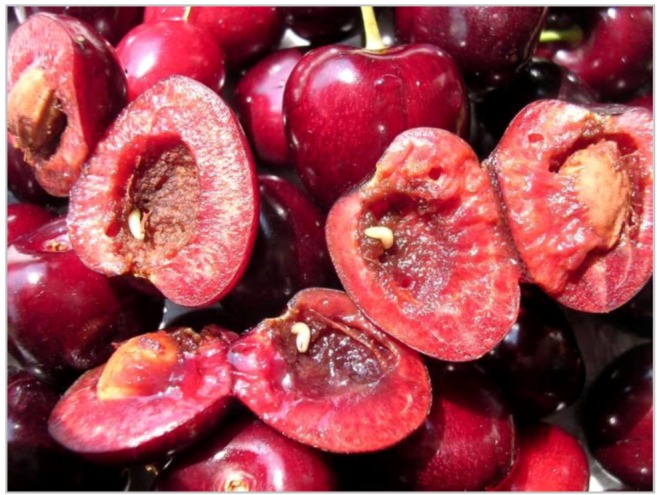
Infested cherries.

**Egg and larval development:** The white eggs have an approximate length of 0.75 mm and a diameter of 0.25 mm [49,59]. Fertility ranges between 54 and 100% [82,94]. A reduced fertility is mainly observed during prolonged periods of fine weather when copulation is reduced in favor of oviposition or after oviposition in unripe cherries [94]. The duration of embryonic development mainly depends on temperature and ranges between two to ten days [7,49,50,58,86]. After eclosion, the larvae immediately move towards the cherry pit in order to find protection from parasitoids and predators [56]. Larval development lasts between 17 [7,50] and 30 days [58], depending on the temperature and the maturity stage of the cherries. The larvae go through three instars, reaching a final size of approximately 6 mm (Figure 3) [95]. During their development, the larvae tunnel in the fruit, macerate the tissue and ingest the broken down pulp [49,58]. Larvae develop better and faster in fruits with higher sugar content and lower acidity [94]. High populations of *R. cerasi *can be therefore observed in sweet cherry orchards, whereas sour cherries usually remain free from high infestations [96,97,98]. 

**Figure 4:**
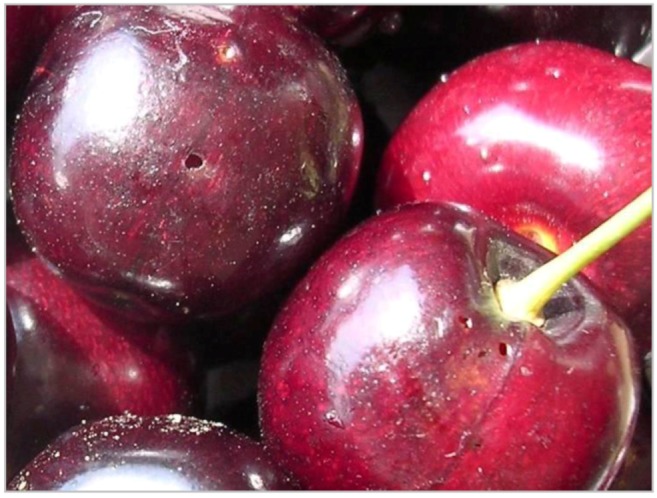
Damaged cherries with exit holes of larvae.

**Figure 5:**
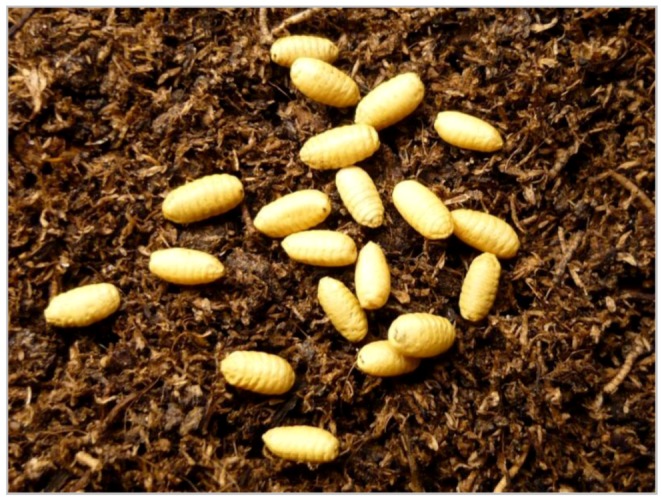
Pupae of *R. cerasi*.

**Pupation:** Around harvest [56], mature larvae bore exit holes through the fruit skin (Figure 4), usually close to the fruit stem [49,99]. Under field conditions, pupation usually occurs within three hours after entering the soil [49]. Most pupae are therefore found directly under the tree canopy, especially under the south and southeast parts of the tree, which is also where the highest fruit infestation levels are observed [100]. Pupation depth is mainly influenced by soil type and usually ranges from 2 to 5 cm [7,56,101,102]. The puparium is straw yellow in color, cylindrical, up to 4 mm long and 2 mm in diameter (Figure 5) [8,49,59].

**Diapause and pupal mortality:** The cherry fruit fly is a univoltine species: The pupae remain in the soil until the following spring. Overwintering pupae enter diapause and require a chilling period before development can continue. Approximately 180 days at temperatures below 5 °C are required for maximum emergence [6,7,38,43,103]. Pupal mortality during the nine to 10 months of diapause is high and is mainly attributed to unfavorable climatic conditions and predation: Usually only 5% [104] to 15% [94] of the pupae emerge in the following year. A few individuals remain in diapause for an additional year or sometimes for several years [6,36,49,50,105]. This pupal carryover is a highly adaptive trait, ensuring that the population will not perish on account of failure of host plants to fruit in some years. However, literature data on the percentage of pupae diapausing for more than one year show wide ranges: from 1 to 21% [101,106], 10% [7,49,105], 7 to 21% [36], 47% [50] and 25 to 100% [107]. A higher percentage remains in diapause for an additional year more frequently in heavy clay soils than in sandy soils [36].

### 3.2. Population Dynamics and Mortality Factors

Many factors (biotic and abiotic) can influence the dynamics of cherry fruit fly populations by directly or indirectly affecting survival and development rates or female fecundity. The most important factors are climatic conditions and host availability. The mortality within one generation can reach 99.6% [94]. However, only a few quantitative studies evaluate the causes of mortality [108]. The basic demographic parameters have been determined by Boller [94]. In cherry production, harvest, and the consequent removal of larvae from the orchard, is considered to be one of the main mortality factors [94]. In addition, temperature and rain have a major impact on mortality. 

Egg and larval stages are well protected inside the cherry. Mortality is generally low during the egg stage [94]. The hatching rate may be reduced when females oviposit in unripe cherries [94]. In addition, some cherry varieties (Schattenmorelle) are known to produce a hard tissue to seclude the eggs [109].

Destruction of cherries by fungal diseases can also lead to increased egg and larval mortality. The first serious cherry fruit fly infestation was observed in Switzerland between 1930 and 1937—it started only three years after a routine treatment of shothole disease (*Stigmina carpophila*) was introduced: Regular yields also lead to improved life conditions for cherry fruit flies [110,111].

Different degrees of infestation are due to phenological differences among cherry varieties and weather conditions during oviposition: Early ripening varieties show lower infestation levels because the fruits are harvested before the first flies are ready to oviposit [47,48,58,98]. Generally, the later a cherry variety is harvested, the higher the potential infestation level [7,98]. Sunny conditions during oviposition lead to high infestation levels [50,102]. Rainy conditions during early ripening stages prevent oviposition and mating [36,49,63,102,112] and might lead to a decay of fruits causing first and second instar larvae to die [110]. However, rainy conditions during harvest, which cause the cherries to crack and the farmers to leave the trees unpicked, might increase the infestation level the following year [7]. Differences in sugar content and acidity of cherry varieties lead to differences in larval nutrition and consequently to differences in fecundity of emerging females [94]. Females from sweet cherry orchards therefore usually show a higher fecundity than females from sour cherry orchards.

The life stages most exposed to climatic conditions and natural enemies are those associated with the soil: mature larvae, pupae and emerging adults. Boller [94] compared the number of larvae dropping from the fruit with the number of pupae in the soil and noted that 35 to 63% of the larvae were not able to pupate because of predation and arid soil conditions. He also monitored the number of pupae in the soil and observed a decline in numbers of pupae during the summer (July, August, September) and during the following spring, which he attributed to predation, parasitism and disease. During emergence, flies are also exposed to different enemies: Boller [94] observed that only 7 to 50% of pupae in the soil during spring produced adult flies. A similar observation was made by Engel [101]: The average number of 147 flies per tree evaluated by treatments with a knockdown insecticide was not consistent with the average number of 9,000 pupae under each tree.

### 3.3. Antagonists of *R. cerasi* and Other Tephritidae

**Viruses:** No literature is available on the effects of viruses on *R. cerasi*. For other Tephritid flies, picornaviruses have been described in *Ceratitis capitata* [113] and in *Bactrocera tryoni* [114]. In addition, reoviruses are known for *Bactrocera oleae* [115,116] and *C. capitata* [117]. No field application strategy has yet been developed for controlling Tephritid flies with viruses.

**Bacteria:** Only few references are available on the use of bacteria to control Tephritid flies, and no references are available for *R. cerasi*. Different isolates of *Bacillus thuringiensis* were screened against larvae and adults of *B. oleae* [118] and *Anastrepha ludens * [119,120]. Endotoxins of different *B. thuringiensis* isolates were tested against adult *C. capitata* [121] and L_3_ larvae of *Anastrepha *sp. [122]. *Bacillus pumilis* was tested against adults and larvae of *C. capitata* in laboratory experiments [123]. In field experiments with four to six applications of *B. thuringiensis* per year against the olive fruit fly *B. oleae*, fruit infestation was reduced by 60% to 80% [124].

**Entomopathogenic fungi:** Many studies have been conducted on the control of *C. capitata*, *Anastrepha fraterculus*, *A. ludens*, *B. oleae* and *B. tryoni* with different entomopathogenic fungi [125,126,127,128,129,130,131,132,133,134,135,136,137,138,139]. Yee and Lacey [140] demonstrated that adult western cherry fruit flies (*R. indifferens*) are susceptible to *Metharizium anisopliae*. Cossentine *et al*. [141] demonstrated that preimaginal *R. indifferens* are susceptible to *Beauveria bassiana*. Until recently, only little was known on fungal pathogens of *R. cerasi*. Wiesmann [49] described adult flies as being susceptible to *Empusa *sp. (Zygomycetes: Entomophthoraceae). In 2009, first evidence was provided that adult *R. cerasi *are susceptible to hyphomycetous fungi [142]. A laboratory screening of different fungus isolates showed that all tested isolates (*B. bassiana*, *M. anisopliae*, *Isaria fumosorosea*, *Isaria farinose*) caused mycosis but virulence varied considerably among the isolates. *B. bassiana* and *I. fumosorosea* caused 90%–100% mortality and had the strongest influence on fecundity. *M. anisopliae* also induced high rates of mortality, while the pathogenicity of *I. farinosa* was low. The effects on L_3_ larvae were tested as well: None of the fungal isolates induced mortality in more than 25% of larvae [142]. These results led to the development of a field application strategy using foliar applications of *B. bassiana* against adult flies [106,143].

**Entomopathogenic nematodes:** Various fruit fly species are known to be susceptible to entomopathogenic nematodes [144,145,146,147,148,149,150]. Yee & Lacey [151] showed good efficacy of *Steinernema* sp. against larvae of the western cherry fruit fly *R. indifferens. *Moreover, recent laboratory studies have indicated promising results of entomopathogenic nematodes to control the third instar larvae of *R. cerasi* [152]. However, results of laboratory experiments conducted in the scope of the European COST 850 project were disappointing: In a screening of 18 different nematode strains, the highest mortality rates in third instar larvae were below 30% (observed after application of *Steinernema feltiae* at a concentration of 1 × 10^5^ infective juveniles m^−2^ on soil, [153]). Field applications of *S. feltiae* and *S. carpocapse* at the rate of 2 × 10^6^ infective juveniles m^−2^ in a cherry orchard in Aesch (BL, northwestern Switzerland) in June 2003 reduced the emergence rate of adults the following year by only 33% (*S. carpocapse*) and 41% (*S. feltiae*), respectively [154]. Similar results (20% reduction of emerging adults) were obtained by Herz *et al*. [104], who conducted field experiments with *S. feltiae* to control *R. cerasi* and noted that the effect of nematodes was masked by high natural pupal mortality during the winter. Due to the limited time frame and the different spatial activity, the potential for entomopathogenic nematodes for controlling *R. cerasi* under field conditions was considered to be rather small. 

**Parasitoids:** Most Tephritid species are attacked by a complex of native parasitoids [145,155]. For *R. cerasi*, 21 species of parasitoids (larval ectoparasitoids, larval endoparasitoids and puparium parasitoids) have been described [156]. No egg parasitoids of *R. cerasi *are mentioned in the literature. In cherry production, however, the effectiveness of larval parasitoids is greatly impaired by the short ovipositor of parasitoid females, which cannot reach *R. cerasi* larvae in large cultivated cherries. Monaco [157] observed that 10 to 30% of *R. cerasi* larvae in wild cherries (*P. mahaleb*) are parasitized by *Utetes *(*Opius) magnus* (Hymenoptera: Braconidae), whereas no parasitization was observed in cultivated cherries. Similar observations were made by Haisch *et al*. [95] and Hoffmeister [66], who noted that *R. cerasi* individuals from *Lonicera *sp. generally showed higher levels of parasitization than individuals from cultivated cherries: *U. magnus* [66] and *Halticoptera laevigata* (Hymenoptera: Pteromalidae) [66,158] have only been observed in individuals from *Lonicera *sp., whereas *Psyttalia *(*Opius) rhagleticola* [66,159] was also found in individuals from cherries—although in lower numbers. Contrary to these observations, Leski [7] showed *P. rhagleticola* to be the principal parasitoid of cherry fruit flies in Poland. However, with parasitization rates of 22 to 32%, *P. rhagleticola* could not control *R. cerasi* populations [7]. Pupal parasitation seems to be more important. *Phygadeuon wiesmanni* (Hymenoptera: Ichneumonidae) occurs throughout Central Europe [94,160,161,162,163,164,165] and has been shown to be responsible for a pupal mortality rate as high as 72% [94,101]. Under bushes of *Lonicera *sp., however*,* the parasitation rates of pupae were found to be higher than under cherry trees [6]. Other puparium parasitoids, such as *Phygadeuon elegans* [165], *Gelis bremeri* (Hymenoptera: Ichneumonidae) [6,7,66], *Polypeza försteri* (Hymenoptera: Diapriidae) [166], and *Spilomicrus hemipterus *(Hymenoptera: Diapriidae) [66], were observed in lower numbers. Until now, no biocontrol strategies based on parasitoids of *R. cerasi* were evaluated under field conditions.

**Predators:** Wiesmann [49] mentions two species of *Odontothrips *sp. (Thysanoptera: Thripidae) attacking the eggs of *R. cerasi*. However, the impact of these predators is considered to be low, as only 10% of the eggs were attacked [49] and as Boller [94] did not observe these predators in his comprehensive studies. Therefore, *R. cerasi* is most likely to be attacked by predators only during the short time span after leaving the fruit and pupation or immediately after emergence. Ants (*Myrmica laevinodis*, Hymenoptera: Formicidae), carabid beetles (*Anisodactylus binotatus*, Coleoptera: Carabidae) or staphylinid beetles (*Paedrus litoralis*, Coleoptera: Staphylinidae) are of particular importance [94,167]. Boller [94] noted that up to 80% of larvae were destroyed by predators before pupation, and that ants seemed to be the most important enemy. According to Boller [94], however, ants are not able to detect and crack the puparia in the soil. This is in contrast to Sajo [168], who observed ants attacking and destroying pupae in the soil. Schwope [169] noted that ants attacked and killed about 40% of the emerging flies. In addition, Boller [94] observed in his experiments that about 15% of pupae were destroyed by small, unidentified organisms, which he believed to be mites.

## 4. History of Cherry Fruit Fly Control

The strategies used to control *R. cerasi* reflect the history of insect control in general. Peaks of research activity for new control strategies coincide with periods of increasing cherry fruit fly populations: The cherry fruit fly usually exhibits four- to five-year periods of high population densities followed by an interval of decline to very low population levels. Boller *et al*. [170] presented the data for Switzerland from 1929 to 1969 and noted that fluctuations in population density were frequently observed throughout Central Europe at the same time. During the first recorded cherry fruit fly outbreak in the 1930s, research mainly focused on bionomics and the behavior of the pest. Initial control methods focused on destruction of infested fruit and the application of inorganic insecticides. During the second wave of high populations in the mid-forties and early fifties, new insecticides (DDT and organophosphorus compounds) were introduced. During the early sixties, the focus shifted toward the development of biotechnical (traps, synthetic host-marking pheromones, and sterile male releases) and biological control methods. Recently, the cherry production is challenged by the withdrawal of insecticides in many countries. The importance of reliable biocontrol strategies is therefore increasing.

### 4.1. Before-Insecticide Strategies—1900 to 1935

Before insecticides were available, farmers knew that an early and complete harvest was the most effective control measure for *R. cerasi* [8,53,56,58,60,171]. Early ripening varieties were recommended for reduced fly damage [53]. The recommendation of eradicating wild and secondary hosts (*Lonicera *sp.) of *R. cerasi* was controversially discussed between Thiem [9] and Wiesmann [172]. However, because the flies from *Lonicera *sp. emerge a few days later than the flies from cherries [42], and because the flies from *Lonicera *sp. show a strong preference for *Lonicera *sp. berries for oviposition [173], it is doubtful whether this recommendation was necessary or justified.

Because *R. cerasi* pupae spend more than 10 months per year in the soil [94] and because the area of pupation is strictly limited to the surface directly under the canopy of infested trees [49], the possibility of soil treatments was appealing [159]. Soil treatments were considered by different authors: Frank [174] suggested soil cultivation in order to bury the pupae more deeply, whereas Mik [8] recommended compression of the soil surface prior to adult emergence. However, according to the results of Thiem [6], a mechanical treatment of the soil surface is not sufficient. He suggested using creosote on larvae shortly before pupation and Tetrachloroethane to kill the pupae. Wiesmann [36] tested a broad range of different means, such as arsenic compounds, naphthalene, dichlorobenzene, nicotine, and kerosene, to control emerging flies or pupae in the soil. He stated that kerosene treatments completely prevented emergence, but that one out of three experimental trees died and another third were badly damaged. Most authors concluded that soil treatments are ineffective to kill the pupae [6,49,53,174,175]. When organo-chemical insecticides such as DDT became available in the 1950s [176], research on soil treatments was abandoned.

### 4.2. First Insecticides Lead Arsenate & DDT—1905 to 1950

The first insecticides—pyrethrum, rotenone, and lead arsenate—were focused on adult flies and were mainly applied in combination with food baits [36,54,60]. However, the efficacy of pyrethrum and rotenone was poor, and lead arsenate was not considered as an option in most European countries due to its high human toxicity [53]. First organo-chemical insecticides such as DDT became available in the 1950s [176] and led to better results in control of adult flies [169,177,178]. However, applications had to be timed exactly to the emergence of flies and repeated treatments were necessary.

### 4.3. Organophosphorus Insecticides—1950 to 2000

With the development and registration of quick-acting organophosphates and carbamates around 1965, a systemic control of eggs and larvae inside the fruit became possible [179,180]. The emphasis of control shifted from the adult to the egg and larval stages. The application date and therefore the flight period became less important. Applications were timed according to the degradation of the various products, as pesticide residues in the harvested crop had to be avoided [181]. Currently, Dimethoate is still in use in some European countries (Table 1), whereas Fenthion is no longer registered because of its high avian toxicity. First attempts to find alternatives to Dimethoate applications were already made in the 1960s.

### 4.4. Research on Population Dynamics and Biotechnical Approaches—1960 to 1990

In order to avoid toxic residues on harvested fruit, great efforts were made to find biological or biotechnical control methods. Different approaches were considered: yellow sticky traps, synthetic host-marking pheromones, and sterile insect technique [14,17,42,170].

**Sticky traps** were developed based on the visual preference of the flies for the color yellow [182]. Remund [75] determined that daylight fluorescent yellow-colored flat surfaces were most attractive. Prokopy [79] suggested that large yellow surfaces represented a super-normal foliage-type stimulus eliciting food-seeking behavior in *R. cerasi* and *R. pomonella*. He also hypothesized that flies reacted to yellow on the basis of true color discrimination. This hypothesis was supported by Agee *et al*. [183], who showed that adult *R. cerasi* had a major peak of electroretinographically assessed spectral sensitivity at 485 to 500 nm (yellow green region) and a secondary peak at 365 nm (ultraviolet region). Traps with a sharp increase of reflectance in the 500 to 520 nm region were found to be the most attractive for *R. cerasi* [183,184]. Based on this knowledge, a three-dimensional wing-shaped trap was developed (Rebell^®^ amarillo) and is now used throughout Europe for monitoring, forecasting and mass trapping purposes [185]. Moreover, mass trapping of flies by Rebell^®^ amarillo became the standard regulation method for *R. cerasi *in organic production. However, in order for mass trapping strategies to be effective, several traps per tree are needed [2]. Remund & Boller [186] suggest using one to eight Rebell^®^ traps, depending on the size of tree, on the southeast side of the canopy. Because the traps should be hung in the upper part of the canopy, much labor is involved, thus making this strategy uneconomical for conventional cherry production (Table 2). 

The use of the **host-marking pheromone** to prevent oviposition was investigated in the 1970s [88,91,187]. In field experiments using naturally derived pheromone, an efficacy of 63 to 90% was observed [187,188]. High synthesis costs, however, prevented the use of this pheromone in commercial cherry growing. In addition, efficacy was low at high infestation levels and under rainy conditions. Moreover, about 10% of the trees had to remain untreated in order to provide unmarked fruits for oviposition [89].

The **sterile insect technique** for cherry fruit fly control was developed between 1960 and 1980 [14,71,189,190,191]. The sterile insect technique is based on the concept that by overflooding natural populations with mass reared, sterilized insects, a high degree of sterility is induced among the eggs produced in the field [170]. Boller [71] could show that the release of sterile males in an isolated 2.5 km^2^ area could reduce infestation below detectable levels. The major bottleneck of this technique is the artificial rearing of the fly [170,192,193,194]. Several points in the insect’s biology complicate rearing: *R. cerasi* is univoltine, has an obligatory diapause of at least 150 days, and *R. cerasi* is monophagous with a strongly selective host choice [88]. The lack of a suitable rearing method for producing enough sterile insects for mass releases prevented this strategy from being commercially introduced.

### 4.5. Development of Biocontrol Strategies—1990 to 2010

Based on first promising laboratory results [152,195], **entomopathogenic nematodes** were considered to be a possible solution for the cherry fruit fly problem. However, field experiments gave disappointing results [104,154] (see Section 3.3). 

The pathogenicity and virulence of different **entomopathogenic fungi** on different life stages of *R. cerasi* were also first evaluated in laboratory experiments. Adult flies were found to be the only life stage susceptible to fungus infection. *B. bassiana* ATCC 74040 showed a high virulence, the flies died during the pre-oviposition period. These results were the first evidence of the susceptibility of *R. cerasi* to infection with hyphomycetous fungi [142]. Field application strategies were therefore focused on adult flies using the fungus isolate *B. bassiana* ATCC 74040, which is formulated in the commercial product Naturalis-L (Intrachem Bio Italia). Repeated applications of Naturalis-L during the flight period of *R. cerasi* were shown to reduce the infestation level of fruits by 60%–70% [143]. The application of Naturalis-L is a suitable and economically reasonable strategy for controlling *R. cerasi* in organic agriculture (Table 2).

In addition to the biocontrol strategies, research on **baits** for possible attract-and-kill-strategies have recently been conducted. Although some of the food baits tested in combination with yellow sticky traps were able to double the number of captured flies [106], none of the baits tested showed economic potential as an effective attract-and-kill system or for mass trapping in commercial production (Table 2). The spinosad GF-120 fruit fly bait (Dow AgroSciences) was tested in several experiments against *R. cerasi* [196]. However, results under humid climate conditions in Switzerland were disappointing. Until now, this strategy is not available for the farmers.

## 5. Currently Used Strategies to Control *R. cerasi*

Until recently, one application of **Dimethoate** was the standard for controlling *R. cerasi* in Swiss sweet cherry production, because it is by far the most cost-efficient method (Table 2)*.* Since 2011, however, this product is no longer registered for use in fruit production in Switzerland because of problems of ecotoxicity and residues on harvested cherries. Two applications of **Acetamiprid** are currently recommended for cherry fruit fly control in Switzerland. The situation in many other European countries is comparable. However, implementation and transition periods differ between the countries. Mainly neonicotinoids and pyrethroids are currently used to control *R. cerasi *(Table 1).

The application of **Naturalis-L** (entomopathogenic fungi *B. bassiana*) is considerably more expensive than the application of Dimethoate or Acetamiprid (Table 2). However, the higher prices obtained for organically grown cherries might justify the higher input for pest control [106]. For good efficacy, four treatments of 0.25% Naturalis-L (5 × 10^4^ CFU mL^−1^) with 1,000 L water per hectare should be applied at seven to ten day intervals. The first application should be made five to ten days after the beginning of the flight period. The time period between the last application and harvest should not exceed seven days. Other phytosanitary measures (early and complete harvest; removal of infested cherries) can further enhance the efficacy of Naturalis-L treatments. Because the use of fungicides can interfere with entomopathogenic fungi, close attention has to be paid to the whole pest management program. In Swiss organic cherry production, only sulfur and neem oil are likely to be applied during the critical period. Fortunately, both pesticides were found to be compatible with entomopathogenic fungi [197,198,199]. However, many of the synthetic fungicides used in integrated pest management strategies were found to be highly toxic to *B. bassiana* [200,201]. Among 36 fungicides tested, only three were compatible with *B. bassiana*, whereas insecticides were less toxic: 24 out of 54 tested insecticides interfered with fungus development [199]. In some cases, differences were found among products containing the same active ingredient (Dimethoate) in different formulations. Thus, the integration of mycoinsecticides for cherry fruit fly control in an organic plant protection system seems possible; including mycoinsecticides into integrated pest management programs might, however, be challenging.

With the increasing number of dwarf tree orchards shielded from rain to prevent the large sized cherry varieties (>24 mm fruit diameter) from splitting, **crop netting** has become a possible method of cherry fruit fly control [202]. Experiments using netting to cover the trees were conducted at the Palatinate Agricultural Service Centre (DLR Rheinpfalz, Germany [203]), at the Bavarian State Research Centre for Agriculture (LfL Bayern, Germany [204]) and at the Research Institute of Organic Agriculture (FiBL, Switzerland, Häseli, personal communication). Available data show that crop netting is a viable, cost-efficient strategy (Table 2) for protecting cherries from infestation. The “Rantai K” net-type with a mesh size of 1.3 mm was used in all experiments. Netting should be installed before the beginning of the flight period and the netting should remain in place until the latest ripening cherry varieties are harvested.

**Covering the soil** under the tree canopy with netting to prevent the hatching flies from reaching the fruit is another efficient management strategy. The netting can reduce fruit infestation by 91% [73]. Because the flies can survive for a long time under the netting, it is advisable to bury the edges of the netting completely. This, however, leads to high labor costs (Table 2). Moreover, expensive, fine-mesh netting (0.8 mm mesh width) is considered to be necessary, because young flies after emergence can easily get through nets with mesh widths of 1.3 mm. Nevertheless, this method could be an option for controlling *R. cerasi* in extensively managed standard tree orchards.

**Mass trapping by yellow sticky traps** is considered to be too expensive for commercial production of cherries (Table 2). Nevertheless, mass trapping may still be the only option for controlling *R. cerasi* in home gardens, in which the application of insecticides is often impossible due to the lack of proper application equipment. Due to the lack of registered alternatives, yellow sticky traps are still widely used in organic cherry production throughout Europe (Table 1). 

**Table 1:** Situation of cherry fruit fly control in different European countries in 2011.

Country harvested area [205]	Management in conventional production	Management in organic production	Reference (personal communication)
Turkey	Cypermethrin	Azadirachtin	T. Koclu &
[35,800 ha]	Delthamethrin	Mass trapping with yellow sticky traps	(Bornova Plant Protection Research Institute)
	Malathion		S. Tezcan
	Methomyl		(Ege University, Bornova)
	Thiacloprid		
Italy	Dimethoate	*Beauveria bassiana*	F. Molinari
[28,900 ha]	Etofenprox	Crop netting	(Università Cattolica del Sacro Cuore, Piacenza)
	Fosmet	Pyrethrum	A. Grassi
	Thiamethoxam	Spinosad	(Istituto Agrario di San Michele all’Adige)
Spain	Lambda-cyhalothrin (bait sprays)	*Beauveria bassiana*	E. Viñuela
[24,671 ha]		Yellow sticky traps	(Universidad Politécnica de Madrid)
Bulgaria	Alpha-cypermethrin	Yellow sticky traps	H. Kutinkova
[11,800 ha]	Bifenthrin		(Fruit Growing Institute, Plovdiv)
	Cypermethrin		
	Deltamethrin		
	Gamma-cyhalothrin		
	Lambda-cyhalothrin		
	Zeta-cypermethrin		
France	Acetamiprid	Yellow sticky traps	S. Simon
[10,752 ha]	Dimethoate		(INRA-UERI Gotheron)
	Deltamethrine		
Greece	Cypermethrin,	*Beauveria bassiana*	B.I. Katsoyannos
[10,000 ha]	Deltamethrin,		(University of Thessaloniki)
	Dimethoate,		
	Thiamethoxam		
Poland	Acetamiprid	Yellow sticky traps	D. Gajek
[9,903 ha]	Pyrethroids	Soil covering	(Agro Research Consulting, Łowicz)
	Thiacloprid		
Portugal	Deltamethrin	Azadirachtin	R. Rodrigues
[6,255 ha]	Dimethoate	Yellow sticky traps	(Escola Superior Agrária de Ponte de Lima*2014Instituto Politécnico de Viana do Castelo)
Germany	No registered insecticide	Use of side effects of pyrethrum applications against aphids	H. Vogt
[5,449 ha]		(Crop netting)	(JKI Dossenheim)
Croatia	Dimethoate	Yellow sticky traps	B. Baric
[3,100 ha]			(Faculty of Agriculture, Zagreb)
Austria	Acetamiprid	Use of side effects of pyrethrum applications against aphids	C. Lethmayer
[2,400 ha]			(AGES Wien)
Hungaria	Acetamiprid	Yellow sticky traps	B. Pénzes
[1,795 ha]	Cypermetrin		(Corvinus University, Budapest)
	Dimethoate		
	Lamda-cyhalotrin		
	Thiachloprid		
	Thiamethoxam		
Albania	Dimethoate	No key pest: no organic strategy	E. Isufi
[1,500 ha]			(Institute for organic Agriculture, Durres)
Belgium	Acetamiprid	Nothing	T. Beliën
[1,224 ha]	Thiacloprid		(PCfruit Belgium)
Switzerland	Acetamiprid	*Beauveria bassiana*	H. Höhn
[454 ha]	Thiachloprid	Crop netting	(agroscope ACW Wädenswil)
	Thiamethoxam	Yellow sticky traps	
	Crop netting		
UK	*R. cerasi* does not occur in the British Isles		J. Cross
[447 ha]			(East Malling Research)
Sweden	No insecticide registered		B. Rämmert
[160 ha]			(Swedish University of Agricultural Sciences, Uppsala)
Slovenia	Acetamiprid	*Beauveria bassiana*	Špela Modic,
[92 ha]	Fosmet	Protein baits	(Agricultural Institute of Slovenia, Ljubljana)

**Table 2:** Costs per hectare of different cherry fruit fly control methods.

	Intensively managed dwarf-tree orchard	Standard trees in semi-intensive systems	Extensively managed standard trees
Trees per ha	800 trees per ha	200 to 500 trees per ha ( 350 trees per ha)	50 to 80 trees per ha ( 65 trees per ha)
Tree size	height of first branches: 0.5 m,	height of first branches: 1.2 m,	height of first branches: 1.8 m,
	tree height: 3.5 m,	tree height: 5 to 6 m,	tree height: 8 to 10 m,
	canopy diameter: 3 to 4 m (7 to 12 m^2^)	canopy diameter: 5 to 7 m (20 to 40 m^2^)	canopy diameter: 11 to 13 m (100 to 130 m^2^)
Dimethoate treatment ^1^	400 L ha^−1^ with 0.8 L Perfekthion^®^, one application: materials: 24.20 € + machines: 50.50 € + labour: 13.42 € **= 88.12****€**	400 L ha^−1^ with 0.8 L Perfekthion^®^, one application: materials: 24.20 € + machines: 50.50 € + labour: 13.42 € **= 88.12****€**	400 L ha^−1^ with 0.8 L Perfekthion^®^, one application: materials: 24.20 € + machines: 50.50 € + labour: 13.42 € **= 88.12****€**
Acetamiprid treatment ^2^	400 L ha^−1^ with 0.32 L kg Gazelle SG, two applications: materials: 184.80 € + machines: 101.00 € + labour: 26.84 € **= 312.64****€**	400 L ha^−1^ with 0.32 L kg Gazelle SG, two applications: materials: 184.80 € + machines: 101.00 € + labour: 26.84 € **= 312.64****€**	400 L ha^−1^ with 0.32 L kg Gazelle SG, two applications: materials: 184.80 € + machines: 101.00 € + labour: 26.84 € **= 312.64****€**
Mass trapping with yellow sticky traps ^3^	One Rebell^®^ trap per tree: materials: 1,812.5 € + labour: 134.19 € **= 1,946.69****€**	Five Rebell^®^ traps per tree: materials: 3,964.84 € + labour:	12 Rebell^®^ traps per tree: materials: 1,767.19 € + labour: 785.00 € **= 2,552.18****€**
		1,761.21 € **= 5,726.05****€**	
Mass trapping with baited yellow sticky traps ^4^	0.5 Rebell^®^ traps per tree with 0.5 TMA-cards: materials: 2,156.25 € + labour: 89.64 € **= 2,245.89****€**	Three Rebell^®^ traps per tree with three TMA-cards: materials: 5,660.17 €+ labour: 1,115.90 € **= 6,776.06****€**	Seven Rebell^®^ traps per tree with seven TMA-cards: materials: 2,452.73 € + labour: 483.56 € **= 2936.29****€**
Soil covering with netting ^5^	materials: 930.75 € + labour: 1,610.25 € **= 2,541.00****€**	materials: 930.75 € + labour: 1,610.25 € **=2,541.00****€**	materials: 930.75 € + labour: 1610.25 € **= 2541.00****€**
Application of Naturalis-L ^6^	800 L ha^−1^ with 2 L Naturalis-L, four applications: materials: 515.00 € + machines: 202.00 € + labour: 53.68 € **= 770.68****€**	1,000 L ha^−1^ with 2.5 L Naturalis-L, four applications: materials 643.75 € + machines: 202.00 € + labour: 53.68 € **= 899.43****€**	Not possible because of insufficient coverage in the upper parts of the canopy
Crop netting ^7^	materials: 242.37 € + labour: 268.38 € **= 510.75****€**	Not possible	Not possible

**Explanatory notes:** Standard costs were calculated according to Arbokost [206], a business management simulation program based on data evaluated in Switzerland. This program is provided by the Federal Research Station agroscope ACW Wädenswil and uses the following values: labor costs 13.42 € per hour; machine costs for pesticide application: 50.50 € per ha and application; time for installation and removal of crop netting 20 hours per ha. For investments: discount rate: 3.5%, amendment factor for discounting 0.6; Costs were calculated using Swiss prices for products. Currency was converted assuming an exchange rate of 1 € = 1.60 CHF.
Perfekthion^®^ (Dimethoate): 30.25 € per L (Leu Gygax AG, Switzerland), 0.8 L ha^−1^, one application. One hour per application per hectare for machine and labor costs. Gazelle SG (Acetamiprid): 288.75 € per kg (Stähler Suisse SA), 0.32 kg ha^−1^, two applications. One hour per application per hectare for machine and labor costs. Rebell^®^ amarillo: 2.27 € per trap (Andermatt Biocontrol AG, Switzerland). Labor input for installation and removal: 45 s per trap (dwarf trees), 4.5 min per trap (in standard tree orchards; estimation made by cherry growers). The traps can be cleaned and re-used: labor input 1 h for 10 traps, material input 9.00 € per 10 traps: 22.42 € per 10 traps = 2.24 € per trap (more or less the same price as new traps). TMA-card: 3.13 € per card (Andermatt Biocontrol AG, Switzerland). Additional time needed to attach the bait to the trap: 15 s per trap. Biocontrol Net 0.8: 0.85 € m^−2^ (Andermatt Biocontrol AG, Switzerland). Because it is not necessary to cover the whole surface, the area covered per ha is reduced to 0.75 ha. Costs for net: 6,375 €; Costs per year (8 years): 930.75 €. Labor input: 120 h (estimated from time needed to set up my experiments). Naturalis-L: 64.38 € per liter (Andermatt Biocontrol AG, Switzerland), 2–2.5 L ha^−1^, four applications. One hour per application per hectare for machine and labor costs. Rantai K: 0.77 € m^−2^ (Hortima AG, Switzerland). Costs for net: 1,291.50 €. Costs per year (6 years): 242.37 €. Assuming that a plastic cover to shelter the fruits against rain is already installed: time for installation and removal of netting: 20 h. Size of net and time needed was calculated according to Balmer [203] and Balmer (personal communication).

## 6. Recommendations for Cherry Fruit Fly Control

Well-managed orchards are a prerequisite for the effective control of *R. cerasi*:

- Trees should be regularly pruned and tree height should be limited to 10 m to allow good coverage of spray applications and to facilitate an early and complete harvest of fruit.
- For new plantings of extensively managed standard trees, varieties suitable for mechanical harvest should be chosen to enable a quick harvest. Harvesting the cherries early and completely reduces the population level of *R. cerasi* by removing the larvae from the orchards before pupation.
- Infested fruits should not be dropped on the ground.
- If possible, early ripening cherry varieties should be chosen, because they mature before the majority of the flies are ready to oviposit.
- It is recommended not to cut the grass under the tree canopies until shortly before harvest. With a higher plant cover, the soil temperatures remain low, which can delay fly emergence for about ten days [207].

Knowledge of first fly appearance is important for a proper timing of control measures. Beginning of the flight period can be determined using forecasting models based on soil temperature measured at a depth of 5 cm. Emergence starts at 430 degree days above the temperature threshold of 5 °C [51,208]. Recently, a forecasting model for *R. cerasi* was included into a database [52] for online presentation and decision support [52]. In addition, depots of pupae in the soil can be used for precise monitoring of emergence [209]. Flight period and flight activity of *R. cerasi* can also be monitored using yellow sticky traps (Rebell^®^ amarillo). In mid-May prior to fly emergence, one or two traps per cherry variety should be placed on the southeast side of the tree canopy in full sun and should be examined twice a week. As long as fly captures remain below a threshold of 0.25 flies per trap in late ripening varieties with an average yield or below one fly per trap in earlier ripening varieties with an outstanding yield, insecticide treatments can be omitted [186]. However, traps are not good indicators of the real infestation level [210]. Depending on yield, weather conditions and trap position, the economic threshold ranges between two and ten flies per trap. Treatment decisions should therefore be based on the expected yield and the infestation level in the previous year. The infestation level can be estimated using the salt solution test [211]: 100 randomly picked cherries of each cherry variety are crushed until the pits are separated from the pulp. A saturated salt solution (350 g salt per liter water) is added. Floating larvae can be counted after 10 min.

Based on economic considerations, the following strategies for cherry fruit fly control are recommended.

- If still registered, one application of Dimethoate at the stage of color change (green to yellow) of cherries is by far the most cost-efficient method.
- Alternatively, Neonicotinoid- or Pyrethroid-products provide a good efficacy with reasonable costs.
- Crop netting with fine-mesh insect net (1.3 mm) to avoid immigration of flies into the orchard provides efficient control in intensively managed dwarf tree orchards covered by plastic or hail net.
- In organic cherry production in orchards without plastic cover or hail net, foliar applications of Naturalis-L (*B. bassiana*) are most suitable.
- The use of yellow sticky traps is very expensive and only reasonable if no other control method is available.

Without the use of systemic insecticides, *R. cerasi* management is still difficult and expensive in extensively managed standard trees. Most of these trees are used to produce cherries for the distillery industry and are not suited to mechanical harvest. Therefore, fruit are usually harvested late, which allows the larvae to pupate in the soil leading to high infestation pressure in the following year. In addition, the grass under the trees is often used for hay or green fodder production. Netting to cover the soil is not always practicable. Mass trapping with traps and baits is expensive, and there are considerable side effects on non-target insects. In addition, cherry growers usually use too few traps per tree, resulting in poor efficacy. Further research is needed to find a strategy for controlling *R. cerasi* in extensively managed standard trees.

## 7. Gaps in Knowledge and Future Research Opportunities

Although during the last 70 years many research projects focused on the development of new regulation strategies for *R. cerasi*, there are still some gaps in knowledge. The following approaches might lead to future regulation methods for *R. cerasi*:

**Mass rearing and release of *Phygadeuon wiesmanni*** (Hymenoptera: Ichneumonidae): This pupal parasitoid has been shown to be responsible for a pupal mortality rate as high as 72% under natural conditions [94,101]. A mass rearing and release of this parasitoid might lead to an effective control of *R. cerasi*. Until now only little effort was made towards this strategy.

**Use of the sexual pheromone**: It was shown that the males produce a highly species-specific pheromone, which attracts females [63,64,65,66,67,68]. Until now this pheromone has not been fully identified. Future work on this topic might lead to more effective traps or confusion technique for *R. cerasi*. 

***Wolbachia*-induced cytoplasmatic incompatibility:** Infestations by different strains of the endosymbiotic bacterium *Wolbachia* lead to a unidirectional cytoplasmatic incompatibility in *R. cerasi* [14,15,19,212,213]. Because *Wolbachia* infestations can profoundly alter host reproduction, research on this topic might lead to new biocontrol approaches of *R. cerasi*.

**Repellents or mechanical barriers to prevent oviposition:** Oviposition behavior of cherry fruit flies is influenced by host fruit characteristics, such as texture [88], surface structure [83], and chemosensory stimuli [88,173,214]. Altering the surface chemistry of cherry fruits might therefore prevent oviposition. Until now only little research has been done on the reaction of *R. cerasi *to non-host volatiles [214,215]. In addition, physical properties of the fruit surface could be altered: It was shown that oil treatments prevent oviposition of *R. cerasi*, because the flies were not able to penetrate the slippery, oily skin with the ovipositor [106]. Residues on harvested fruit were a drawback with oil applications. However, mechanical barriers seem promising.

## References

[1] Fimiani P., Cavalloro R. (1983). Multilarval infestations by *Rhagoletis cerasi* L. (Diptera: Trypetidae) in cherry fruits. Fruit Flies of Economic Importance.

[2] Boller E. (1972). Zum Verkauf und Einsatz neuer Kirschenfliegenfallen im Jahre 1972. Schweiz. Z. Obst-und Weinbau.

[3] Headrick D.H., Goeden R.D. (1998). The biology of nonfrugivorous tephritid fruit flies. Annu. Rev. Entomol..

[4] White I.M., Elson-Harris M.M. (1992). Fruit Flies of Economic Significance: Their Identification and Binomics.

[5] Boller E., Prokopy R.J. (1976). Bionomics and management of *Rhagoletis*. Annu. Rev. Entomol..

[6] Thiem H. (1934). Beiträge zur Epidemiologie und Bekämpfung der Kirschfruchtfliege (*Rhagoletis cerasi* L.). Arb. Physiol. Angew. Entomol. Berl. Dahlem.

[7] Leski R. (1963). Studia nad biologia i ecologia nasionnicy tzresniowki *Rhagoletis cerasi* L. (Diptera: Trypetidae). Pol. Pismo Entomol. Ser. B.

[8] Mik J. (1898). Zur Biologie von Rhagoletis cerasi L. nebst einigen Bemerkungen über die Larven und Puparien der Trypetiden und über die Fühler der Musciden-Larven. Wien. Entomol. Ztg..

[9] Thiem H. (1939). Über die Bedeutung der wilden Wirtspflanzen der Kirschfruchtfliege (*Rhagoletis cerasi* L.) für die Verbreitung und Bekämpfung des Schädlings. Arb. Physiol. Angew. Entomol. Berl. Dahlem.

[10] Ranner H. (1987). Untersuchungen zur Biologie und Bekämpfung der Kirschfruchtfliege, *Rhagoletis cerasi* L. (Diptera, Trypetidae)-I. Die Bedeutung der wilden Wirtspflanzen für die Epidemiologie und die ökologische Differenzierung der Kirschfruchtfliege. Pflanzenschutzberichte.

[11] Wiesmann R. (1938). Befällt die Kirschfliege ausser der Kirsche auch andere Wirte und welche Bedeutung haben diese Wirte. Schweiz. Z. Obst-und Weinbau.

[12] Thiem H. (1932). Heckenkirschen und Sauerdorn als Wirtspflanzen der Kirschfruchtfliege (*Rhagoletis cerasi *L.). Nachrichtenbl. Dtsch. Pflanzenschutzd..

[13] Jaastad G. (1994). First registration of the Cherry Fruit Fly, *Rhagoletis cerasi* (L.) in western Norway: Distribution, size and origin of the population. Nor. J. Agric. Sci..

[14] Boller E., Russ K., Vallo V., Bush G.L. (1976). Incompatible races of European Cherry Fruit Fly, *Rhagoletis cerasi* (Diptera-Tephritidae), their origin and potential use in biological control. Entomol. Exp. Appl..

[15] Riegler M., Stauffer C. (2002). Wolbachia infections and superinfections in cytoplasmically incompatible populations of the European cherry fruit fly *Rhagoletis cerasi* (Diptera, Tephritidae). Mol. Ecol..

[16] Boller E., Robinson A.S., Hooper G. (1989). Cytoplasmatic incompatibility in *Rhagoletis cerasi*. Fruit Flies Their Biology, Natural Enemies and Control.

[17] Matolin S. (1976). Mechanism causing incompatibility between different strains of *Rhagoletis cerasi* (Diptera, Tephritidae). Acta Entomol. Bohemoslov..

[18] Ranner H. (1988). Untersuchungen zur Biologie und Bekämpfung der Kirschfruchtfliege, *Rhagoletis cerasi* L. (Diptera, Trypetidae)-IV. Statistiche Auswertung von Kreuzenversuchen mit Kirschfliegen verschiedenen Alters and Puppengewichts, verschiedener Wirtspflanzenherkunft und Rassenzugehörigkeit. Pflanzenschutzberichte.

[19] Blümel S., Keck M., Nowotny N., Fiedler W., Russ K. (1991). Nachweis und Therapie von Rickettsia-like organisms (RLO’s) in den Ovarien der Europäischen Kirschfruchtfliege (*Rhagoletis cerasi* L.; Trypetidae): Ein Beitrag zur Frage der unidirektionalen Kreuzungssterilitat dieser Art. Pflanzenschutzberichte.

[20] Riegler M. (2002). Der Endosymbiont Wolbachia (alpha-Proteobacteria) in der europäischen Kirschfruchtfliege *Rhagoletis cerasi* (Diptera, Tephritidae): Populationsdynamik und Einfluss auf die Populationsgenetik. Ph.D. thesis.

[21] EPPO (2003). *Rhagoletis cingulata* n'est pas present en Allemagne.

[22] EPPO (2004). *Rhagoletis cingulata* occurs in the Netherlands, but not *R. indifferens*.

[23] EPPO (2006). Distribution maps of quarantine pests for Europe-*Rhagoletis cingulata*.

[24] Smith I.M., McNamara D.G., Scott P.R., Holderness M., EPPO (1996). CABI *Rhagoletis cingulata* and *Rhagoletis indifferens*. Quarantine pests for Europe;.

[25] Egartner A., Zeisner N., Hausdorf H., Blümel S. (2010). First record of *Rhagoletis cingulata* (Loew) (Dipt., Tephritidae) in Austria. EPPO Bull..

[26] Boller E., Mani E. (1994). Two North American *Rhagoletis *spp. in Europe. IOBC/WPRS Bull..

[27] Boller E. (2000). Situationsbericht über nordamerikanische Fruchtfliegenarten in der Schweiz (Diptera: Tephritidae).

[28] Lampe I., Burghause F., Krauthausen H.J. Introduction and distribution of the American cherry fruit fly Rhagoletis cingulata in the Rhine Valley, Germany. Proceedings of the Plant Protection and Plant Health in Europe.

[29] Lampe I., Dahlbender W., Harzer U., Hensel G., Krauthausen H.J. (2006). Die Amerikanische Kirschfruchtfliege (*Rhagoletis cingulata*)-Untersuchungen zum Auftreten in Rheinland-Pfalz. Obstbau.

[30] Vogt H., Köppler K., Dahlbender W., Hensel G. Observations of Rhagoletis cingulata, an invasive species from North America, on cherry in Germany. Proceedings of the International Conference on Integrated Fruit Production, IOBC.

[31] Zwölfer H., Cavalloro R. (1983). Life systems and strategies of ressource exploitation in tephritids. Fruit Flies of Economic Importance.

[32] Moraiti C.A., Nakas C.T., Papadopoulos N.T.  (2012). Prolonged pupal dormancy is associated with significant fitness cost for adults of *Rhagoletis cerasi* (Diptera: Tephritidae). J. Insect Physiol..

[33] Bateman M.A. (1972). The ecology of fruit flies. Annu. Rev. Entomol..

[34] Fletcher B.S., Robinson A.S., Hooper G. (1989). Life history strategies of tephritid fruit flies. Fruit Flies Their Biology,Natural Enemies and Control..

[35] Sivinski J., Burk T., Robinson A.S., Hooper G. (1989). Reproductive and mating behavior. Fruit Flies Their Biology,Natural Enemies and Control..

[36] Wiesmann R. (1934). Untersuchungen über die Lebensgeschichte und Bekämpfung der Kirschenfliege *Rhagoletis cerasi* Linné-II. Mitteilung. Landw. Jahrb. Schweiz..

[37] Kovanci O.B., Kovanci B. (2006). Effect of altitude on seasonal flight activity of *Rhagoletis cerasi* flies (Diptera: Tephritidae). Bull. Entomol. Res..

[38] Haisch A. (1975). Zur Puppendiapause der Kirschenfliege *Rhagoletis ceras*i L.-I. Beeinflussung der diapausierenden Puppen durch unterschiedliche Temperaturen und verschieden lange Kälteexpositionen. J. Appl. Entomol..

[39] Wiesmann R. (1950). Untersuchungen über die Diapause der Puppe der Kirschenfliege *Rhagoletis cerasi* L. (Dipt. Trypetid.). Mitt. Schweiz. Entomol. Ges..

[40] Haisch A., Chwala D. (1979). ber den Einfluss wechselnder Temperaturen auf den Diapause-Ablauf der Europäischen Kirschfruchtfliege *Rhagoletis cerasi* [Diptera, Trypetidae]. Entomol. Gen..

[41] Ranner H. (1988). Untersuchungen zur Biologie und Bekämpfung der Kirschfruchtfliege, *Rhagoletis cerasi* L. (Diptera, Trypetidae)-III. Statistischer Vergleich der Schlupfperioden und Schlupfraten der Kirschfliege. Pflanzenschutzberichte.

[42] Boller E., Bush G.L. (1974). Evidence for genetic variation in populations of the European Cherry Fruit Fly, *Rhagoletis cerasi* (Diptera: Tephritidae) based on physiological parameters and hybridization experiments. Entomol. Exp. Appl..

[43] Haisch A., Forster S. (1975). Zur herkunftsspezifischen Diapause-Ausprägung der Europäischen Kirschfruchtfliege, *Rhagoletis cerasi* (Diptera: Trypetidae). Entomol. Ger..

[44] Thiem H. (1940). Über die Bedeutung der wilden Wirtspflanzen der Kirschfruchtfliege (*Rhagoletis cerasi* L.) für die Verbreitung und Bekämpfung des Schädlings. Arb. Physiol. Angew. Entomol. Berl. Dahlem.

[45] Baker C.R.B., Miller G.W. (1978). Effect of temperature on postdiapause development of 4 geographical populations of European Cherry Fruit Fly (*Rhagoletis cerasi*). Entomol. Exp. Appl..

[46] Papanastasiou S.A., Nestel D., Diamantidis A.D., Nakas C.T., Papadopoulos N.T. (2011). Physiological and biological patterns of a highland and a coastal population of the European cherry fruit fly during diapause. J. Insect Physiol..

[47] Böhm H. (1949). Untersuchungen über die Lebensweise und Bekämpfung der Kirschfliege (*Rhagoletis cerasi* L.). Pflanzenschutzberichte.

[48] Jancke O., Böhmel W. (1933). Beitrag zur Biologie und Bekämpfung der Kirschenfliege. Arb. Biol. Reichsanstalt.

[49] Wiesmann R. (1933). Untersuchungen über die Lebensgeschichte und Bekämpfung der Kirschenfliege *Rhagoletis cerasi* Linné-I. Mitteilung.Landw. Jahrb. Schweiz..

[50] Thiem H. (1935). Untersuchungen zur Biologie der Kirschfruchtfliege (*Rhagoletis cerasi* L.) und ihrer Wirtspflanzen. Arb. Physiol. Angew. Entomol. Berl. Dahlem.

[51] Boller E.  (1964). Auftreten der Kirschenfliege (*Rhagoletis cerasi* L.) und Prognose mittels Bodentemperaturen im Jahre 1963. Schweiz. Z. Obst-und Weinbau.

[52] Samietz J., Graf B., Höhn H., Schaub L., Höpli H.U. (2007). Phenology modelling of major insect pests in fruit orchards from biological basics to decision support: The forecasting tool SOPRA. EPPO Bull..

[53] Sprengel L. (1932). Biologische und epidemiologische Untersuchungen als Grundlage für die Bekämpfung der Kirschfruchtfliege *Rhagoletis cerasi* L. Gartenbauwissenschaft.

[54] Berlese A. (1906). Probabile methodo di lotta efficace contro la *Ceratitis capitata*, *Rhagoletis cerasi* ed altri Tripetidi. Redia.

[55] Wiesmann R. (1935). Ergebnisse dreijähriger Untersuchungen über die Biologie und Bekämpfung der Kirschfliege *Rhagoletis cerasi* L. in der Schweiz. Anz. Schadl. J. Pest Sci..

[56] Wiesmann R. (1934). Die Kirschfliege und ihre Bekämpfung. Schweiz. Z. Obst-und Weinbau.

[57] Verguin J. (1927). La mouche des cerises (*Rhagoletis cerasi*)-Etat actuel de la question. Ann. Epiphyties.

[58] Verguin J. (1928). La mouche de cerises, *Rhagoletis cerasi* L.: Sa biologie, les moyens de la combattre. Rev. Zool. Agricole Appl..

[59] Samoggia A. (1932). Nota sulla *Rhagoletis cerasi* L. Boll. Lab. Entomol. R. Inst. Super. Agrar..

[60] Sprengel L. (1932). Bekämpfung der Kirschfruchtfliege (*Rhagoletis cerasi* L.). Anz. Schadl. J. Pest Sci..

[61] Wiesmann R. (1944). Untersuchungen über das Anködern der Kirschfliege *Rhagoletis cerasi* L. Landw. Jahrb. Schweiz..

[62] Stamenkovic S., Milenkovic S., Stamenkovic T., Hampson C.R., Anderson R.L., Perry R.L., Webster A.D. (1996). Population dynamics of *Rhagoletis cerasi* L. (Diptera, Tephritidae) in western Serbia. Act. Hortic..

[63] Katsoyannos B.I. (1979). Zum Reproduktions und Wirtswahlverhalten der Kirschenfliege, *Rhagoletis cerasi* L. (Diptera: Tephritidae). Ph.D. (Thesis no. 6409) thesis.

[64] Katsoyannos B.I. (1982). Male sex pheromone of *Rhagoletis cerasi* L. (Diptera, Tephritiidae): Factors affecting release and response and its role in mating behavior. J. Appl. Entomol..

[65] Katsoyannos B.I. (1976). Female attraction to males in*Rhagoletis cerasi* (Diptera-Tephritidae). Environ. Entomol..

[66] Hoffmeister T. (1992). Aspekte der Partnerfindung, Konkurrenz und Parasitierung frugivorer Bohrfliegen (Diptera: Tephritidae)Christian Albrechts Universität. Ph.D. thesis.

[67] Raptopoulos D., Haniotakis G., Koutsaftikis A., Kelly D., Mavraganis V. (1995). Biological activity of chemicals identified from extracts and volatiles of male *Rhagoletis cerasi*. J. Chem. Ecol..

[68] Katsoyannos B.I., Robinson A.S., Hooper G. (1989). Mating Pheromones-*Rhagoletis* ssp. Fruit Flies Their Biology,Natural Enemies and Control.

[69] Katsoyannos B.I., Boller E., Benz G. (1986). Das Verhalten der Kirschenfliege, *Rhagoletis cerasi*, L., bei der Auswahl der Wirtspflanzen und ihre Dispersion. Mitt. Schweiz. Entomol. Ges..

[70] Boller E. (1969). Neues über die Kirschenfliege: Freilandversuche im Jahr 1969. Schweiz. Z. Obst-und Weinbau.

[71] Boller E., Remund U., Cavalloro R. (1983). Field Feasibility Study for the Application of SIT in *Rhagoletis cerasi* L. in Northwest Switzerland (1976-1979). Fruit Flies of Economic Importance.

[72] Remund U., Boller E. (1975). Qualitätskontrolle von Insekten: Die Messung von Flugparametern. Z. Angew. Entomol..

[73] Daniel C., Wyss E., Mayer J., Alföldi T., Leiber F., Dubois D., Fried P., Heckendorn F., Hillmann E., Klocke P., Lüscher A., Riedel S. (2009). Migration und Ausbreitung der Kirschfruchtfliege innerhalb von Obstanlagen**-**Möglichkeit der biologischen Bodenbehandlung. Proceedings of the 10th Wissenschaftstagung Ökologischer Landbau.

[74] Prokopy R.J. (1969). Visual responses of European cherry fruit flies-*Rhagoletis cerasi* L. (Diptera, Trypetidae). Pol. Pismo Entomol..

[75] Remund U. (1971). Anwendungsmöglichkeiten einer wirksamen visuellen Wegwerffalle für die Kirschenfliege. Schweiz. Z. Obst-und Weinbau.

[76] Haisch A., Forster S. (1969). Versuche zur Anköderung und zum Fang der Kirschenfliege (*Rhagoletis cerasi* L.). Anz. Schadl. J. Pest Sci..

[77] Haisch A., Forster S. (1970). Erfahrungen beim Fang der Kirschenfliege mit Leimtafeln und Leimkugeln. Gesunde Pflanz.

[78] Katsoyannos B.I., Robinson A.S., Hooper G. (1989). Response to Shape, Size and Color. Fruit Flies Their Biology, Natural Enemies and Control.

[79] Prokopy R.J. Orientation of the apple maggot flies *Rhagoletis pomonella* (Walsh) and European cherry fruit flies* R. cerasi* L. (Diptera: Tephritidae) to visual stimuli. Proceedings of the 13 International Congress of Entomology.

[80] Städler E., Schöni R. (1991). High sensitivity to sodium in the sugar chemoreceptor of the cherry fruit fly after emergence. Physiol. Entomol..

[81] Katsoyannos B.I., Boller E., Benz G. (1987). Zur Reproduktionsbiologie der Kirschenfliege *Rhagoletis cerasi* L.: Präovipositionsperiode, Tagesperiodizität und Einfluss der Kopulation auf die Fekundität und Fertilität einzeln oder in Gruppen gehaltener Weibchen (Diptera: Tephritidae). Mitt. Schweiz. Entomol. Ges..

[82] Boller E. (1966). Beitrag zur Kenntnis der Eiablage und Fertilität der Kirschenfliege *Rhagoletis cerasi* L. Mitt. Schweiz. Entomol. Ges..

[83] Wiesmann R. (1937). Die Orientierung der Kirschfliege, *Rhagoletis cerasi* L., bei der Eiablage. Landw. Jahrb. Schweiz..

[84] Boller E. (1968). An artifical oviposition device for European Cherry Fruit Fly *Rhagoletis cerasi*. J. Econ. Entomol..

[85] Boller E.F., Robinson A.S., Hooper G. (1989). Rearing-*Rhagoletis* spp. Fruit Flies Their Biology,Natural Enemies and Control.

[86] Sprengel L., Sonntag K. (1932). Der Flug der Kirschfliege (*Rhagoletis cerasi* L.) in seiner Bedeutung zu Fruchtreife und Witterung, mit grundsätzlichen Erörterungen über die Erfassung der Wetterfaktoren. Anz. Schadl. J. Pest Sci..

[87] Häfliger E. (1953). Das Auswahlvermögen der Kirschenfliege bei der Eiablage. Mitt. Schweiz. Entomol. Ges..

[88] Katsoyannos B.I. (1975). Oviposition-deterring, male-arresting, fruit-marking pheromone in *Rhagoletis cerasi*. Environ. Entomol..

[89] Aluja M., Boller E.F. (1992). Host marking pheromone of *Rhagoletis cerasi*: Field deployment of synthetic pheromone as a novel cherry fruit fly management strategy. Entomol. Exp. Appl..

[90] Boller E., Aluja M. (1992). Oviposition deterring pheromone in *Rhagoletis cerasi* L. J. Appl. Entomol..

[91] Hurter J., Boller E., Städler E., Raschdorf F., Schreiber J., Cavalloro R. (1989). Oviposition-deterring pheromone in *Rhagoletis cerasi* L. Purification and determination of the chemical constitution. Fruit Flies of Economic Importance.

[92] Dederichs U. (2003). Südbaden: Probleme bei der Kirschenfliegenbekämpfung. Obstbau.

[93] Fimiani P. (1984). Ricerche bioecologiche sulla mosca della ciliege (*Rhagoletis cerasi* L.) in Campania. II. Distribuzione e intensita delle infestazioni larvali. Boll. Lab. Entomol. Agrar. Filippo Silvestri.

[94] Boller E. (1966). Der Einfluss natürlicher Reduktionsverfahren auf die Kirschenfliege *Rhagoletis cerasi* L. in der Nordwestschweiz, unter besonderer Berücksichtigung des Puppenstadiums. Schweiz. Landw. Forsch..

[95] Haisch A., Boller E., Russ K., Vallo V., Fimiani P. (1978). The European Cherry Fruit Fly (*Rhagoletis cerasi* Linné 1758) synopsis and bibliographie. IOBC/WPRS Bull..

[96] Balazs K., Jenser G.  (2004). Significance of the parasitoids and predators in IPM of sour-cherry. IOBC/WPRS Bull..

[97] Fimiani P., Frilli F., Inserra S., Monaco R., Sabatino A. (1981). Ricerche coordinate su aspetti bioecologici della *Rhagoletis cerasi* in Italia. Boll. Lab. Entomol. Agrar. Filippo Silvestri.

[98] Stamenkovic S., Stamenkovic T., Milenkovic S., Nicolic M. (1996). Susceptibility of some sweet cherry cultivars to *Rhagoletis cerasi* L. (Diptera, Tephritidae). Act. Hortic..

[99] Sprengel L. (1932). Kirschfruchtfliege und ihre Bekämfung.

[100] Engel H. (1969). Versuche zur Bekämpfung der Kirschfruchtfliege mit Leimtafeln. Gesunde Pflanz.

[101] Engel H. (1976). Untersuchungen über die Besatzdichte der Kirschfruchtfliege (*Rhagoletis cerasi* L.). Z. Pflanzenk Pflanz..

[102] Ménegaux R. (1898). Le ver de cerises. Le Naturaliste.

[103] Vallo V., Remund U., Boller E. (1976). Storage conditions of stock-piled daipausing pupae of *Rhagoletis cerasi* for obtaining high emergence rates. Entomophaga.

[104] Herz A., Köppler K., Vogt H., Zikeli S., Claupein W., Dabbert S., Kaufmann B., Müller T., Valle Zárate A. (2007). Kann der Einsatz entomopathogener Nematoden zur nachhaltigen Bekämpfung der Kirschfruchtfliege beitragen?. Proceedings of the 9. Wissenschaftstagung Ökologischer Landbau.

[105] Speyer W. (1941). Beobachtungen über das Zahlenverhältnis der Geschlechter bei der Kirschfruchtfliege (*Rhagoletis cerasi* L.). Z. Pflanzenk Pflanz..

[106] Daniel C. (2009). Entomopathogenic fungi as a new strategy to control the European cherry fruit fly *Rhagoletis cerasi* Loew (Diptera: Tephritidae). Ph.D. thesis.

[107] Sajo K. (1902). Zur Entwicklung der Kirschfliege. Prometheus.

[108] Boller E., Remund U., Cavalloro L. (1989). Qualitative and quantitative life-table studies in *Rhagoletis cerasi* L. in Nothwest Switzerland. Fruit Flies of Economoc Importance.

[109] Thiem H. (1954). Wie ernte ich madenfreie Kirschen.

[110] Wiesmann R.  (1943). Neue Untersuchungen über die Bekämpfung der Kirschenfliege, *Rhagoletis cerasi* L. Schweiz. Z. Obst-und Weinbau.

[111] Meier K.  (1932). Das Auftreten der Kirschenfliege. Schweiz. Z. Obst-und Weinbau.

[112] Prokopy R.J., Boller E. (1971). Stimuli eliciting oviposition of European Cherry Fruit Flies, *Rhagoletis cerasi* (Diptera: Tephritidae), into inanimate objects. Entomol. Exp. Appl..

[113] Plus N., Cavalloro R., Cavalloro R. (1983). The viruses of *Ceratitis capitata *Wied. *in vivo* and *in vitro*. Fruit Flies of Economic Importance.

[114] Bashiruddin J.B., Martin J.L., Reinganum C. (1988). Queensland fruit fly virus, a probable member of Picornaviridae. Arch. Virol..

[115] Anagnou-Veroniki M., Veyrunes C.J., Kuhl G., Bergoin M. (1997). A nonoccluded reovirus of the olive fly, *Dacus oleae*. J. Gen. Virol..

[116] Manousis T., Moore N.F., Cavalloro R. (1989). The Serach for Viruses Pathogenic for the Olive Fruit Fly, Dacus oleae Gmelin. Fruit Flies of Economic Importance.

[117] Plus N., Cavalloro R. (1989). The Reoviruses of Trypetidae, Drosophilidae and Muscidae-A Review. Fruit Flies of Economic Importance.

[118] Alberola T.M., Aptosoglou S., Arsenakis M., Bel Y., Delrio G., Ellar D.J., Ferre J.,  Granero F., Guttmann D.M., Koliais S. (1999). Insecticidal activity of strains of *Bacillus thuringiensis* on larvae and adults of *Bactrocera oleae* Gmelin (Dipt. Tephritidae). J. Invertebr. Pathol..

[119] Robacker D.C., Martinez J.A., Garcia J.A., Diaz M., Romero C. (1996). Toxicity of *Bacillus thuringiensis* to Mexican fruit fly (Diptera: Tephritidae). J. Econ. Entomol..

[120] Martinez A.J., Robacker D.C., Garcia J.A. (1997). Toxicity of an isolate of *Bacillus thuringiensis* subspecies *darmstadiensis* to adults of the Mexican fruit fly (Diptera: Tephritidae) in the laboratory. J. Econ. Entomol..

[121] Gingrich R.E. (1987). Demonstration of *Bacillus thuringiensis* as a potential control agent for the Mediterranean fruit fly *Ceratitis capitata *(Wied.). J. Appl. Entomol..

[122] Toledo J. (1999). Toxicity of *Bacillus thuringiensis* beta-endotoxin to three species of fruit flies (Diptera: Tephritidae). J. Econ. Entomol..

[123] Molina C.A., Caña-Roca J.F., Osuna A., Vilchez S. (2010). Selection of a* Bacillus pumilus* strain highly active against *Ceratitis capitata* (Wiedemann) larvae. Appl. Environ. Microb..

[124] Navrozidis E.I., Vasara E., Karamanlidou G., Salpiggidis G.K., Koliais S.I. (2000). Biological control of *Bactocera oleae* (Diptera: Tephritidae) using a Greek *Bacillus thuringiensis* isolate. J. Econ. Entomol..

[125] Mochi D.A., Monteiro A.C., de Bortoli S.A., Doria H.O.S., Barbosa J.C. (2006). Pathogenicity of *Metarhizium anisopliae* for *Ceratitis capitata* (Wied.) (Diptera: Tephritidae) in soil with different pesticides. Neotrop. Entomol..

[126] Garcia A.T.E., Souza H.M.L.D., Messias C.L., Piedrabuena A.E. (1989). Patogenicidade de *Metarhizium anisopliae* nas diferentes fases de desenvolvimento de *Ceratitis capitata* (Wied.) (Diptera, Tephritidae). Rev. Bras. Entomol..

[127] Ekesi S., Maniania N.K., Mohamed S.A., Lux S.A. (2005). Effect of soil application of different formulations of *Metarhizium anisopliae* on African tephritid fruit flies and their associated endoparasitoids. Biol. Control.

[128] Dimbi S., Maniania N.K., Lux S.A., Mueke J.M. (2004). Effect of constant temperatures on germination, radial growth and virulence of *Metarhizium anisopliae* to three species of African tephritid fruit flies. BioControl.

[129] Castillo M.A., Moya P., Hernándeza E., Primo-Yúferab E. (2000). Susceptibility of *Ceratitis capitata* Wiedemann (Diptera: Tephritidae) to entomopathogenic fungi and their extracts. Biol. Control.

[130] Queseda-Moraga E., Ruiz-Garcia A., Santiago-Alvarez C. (2006). Laboratory evaluation of entomopathogenic fungi *Beauveria bassiana *and *Metarhizium anisopliae* against puparia and adults of *Ceratitis capitata* (Diptera: Tephritidae). J. Econ. Entomol..

[131] Lezama-Gutierrez R., Trujillo-De la Luz A., Molina-Ocha J., Rebolledo-Dominguez O., Pescador A.R., Lopez-Edwards M., Aluja M. (2000). Virulence of *Metarhizium anisopliae* on *Anastrepha ludens*: Laboratory and field trials. J. Econ. Entomol..

[132] De La Rosa W., Lopez F.L., Liedo P. (2002). *Beauveria bassiana* as a pathogen of the mexican fruit fly (Diptera: Tephritidae) under laboratory conditions. J. Econ. Entomol..

[133] Toledo J., Campos S.E., Flores S., Liedo P., Barrera J.F., Villaseñor A., Montoya P. (2007). Horizontal transmission of *Beauveria bassiana* in *Anastrepha ludens *(Diptera: Tephritidae) under laboratory and field cage conditions. J. Econ. Entomol..

[134] Carneiro R.M.D.G., Salles L.A.B. (1994). Patogenicidade de *Paecilomyces fumosoroseus*, isolado CG 260 sobre larvas e pupas de *Anastrepha fraterculus* Wied. An Soc. Entomol. Bras..

[135] Destefano R.H.R., Bechara I.J., Messias C.L., Piedrabuena A.E. (2005). Effectiveness of *Metarhizium anisopliae* against immature stages of *Anastrepha fraterculus* fruitfly (Diptera: Tephritidae). Braz. J. Microb..

[136] Konstantopoulou M.A., Mazomenos B.E.  (2005). Effectiveness of *Metarhizium anisopliae* against immature stages of *Anastrepha fraterculus* fruitfly (Diptera: Tephritidae). Braz. J. Microb..

[137] Anagnou-Veroniki M., Kontodimas D.C., Adamopoulos A.D., Tsimboukis N.D., Voulgaropoulou A. (2005). Effects of two fungal based biopesticides on *Bactrocera (Dacus) oleae *(Gmelin) (Diptera: Tephritidae). IOBC/WPRS Bull..

[138] Carswell I., Spooner-Hart R., Milner R.J. (1998). Laboratory susceptibility of *Musca domestica* L. (Diptera: Muscidae) and *Bactrocera tryoni* (Frogatt) (Diptera: Tephritidae) to an isolate of *Metarhizium anisopliae* (Metsch.) Sorokin. Aust. J. Entomol..

[139] Garrido-Jurado I., Torrent J., Barrón V., Corpas A., Quesada-Moraga E. (2011). Soil properties affect the availability, movement, and virulence of entomopathogenic fungi conidia against puparia of *Ceratitis capitata* (Diptera: Tephritidae). Biol. Control.

[140] Yee W.L., Lacey L.A. (2005). Mortality of different life stages of *Rhagoletis indifferens* (Diptera: Tephritidae) exposed to the entomopathogenic fungus *Metarhizium anisopliae*. J. Entomol. Sci..

[141] Cossentine J., Thistlewood H., Goettel M., Jaronski S.T. (2010). Susceptibility of preimaginal western cherry fruit fly, *Rhagoletis indifferens* (Diptera:Tephritidae) to *Beauveria bassiana* (Balsamo) Vuillemin Clavicipitaceae (Hypocreales). J. Invertebr. Pathol..

[142] Daniel C., Wyss E. (2009). Susceptibility of different life stages of the European Cherry Fruit Fly, *Rhagoletis cerasi*, to entomopathogenic fungi. J. Appl. Entomol..

[143] Daniel C., Wyss E. (2010). Field applications of *Beauveria bassiana* to control the European Cherry Fruit Fly *Rhagoletis cerasi*. J. Appl. Entomol..

[144] Gazit Y., Rössler Y., Glazer I. (2000). Evaluation of entomopathogenic nematodes for the control of mediteranean fruit fly. Biocontrol Sci. Technol..

[145] Gingrich R.E., Aluja M., Liedo P. (1993). Biological Control of Tephritid Fruit Flies by Inundative Releases of Natural Enemies. Fruit Flies: Biology and Management.

[146] Lindgren J.E., Vail P.V. (1986). Response of Mediteranean fruit fly, Melon fly and Oriental fruit fly (Diptera:Tephritidae) to the entomogenous nematode *Steinernema feltiae* in field tests in Hawaii. Environ. Entomol..

[147] Lindgren J.E., Vail P.V. (1986). Susceptibility of Mediteranean fruit fly, Melon fly and Oriental fruit fly (Diptera:Tephritidae) to the entomogenous nematode *Steinernema feltiae* in laboratoy tests. Environ. Entomol..

[148] Patterson Stark J.E., Lacey L.A. (1999). Susceptibility of Western cherry fruit fly (Diptera: Tephritidae) to five species of entomopathogenic nematodes in laboratory studies. J. Invertebr. Pathol..

[149] Toledo J., Ibarra J.E., Liedo P., Gomez A., Rasgado M.A., Williams T. (2005). Infection of *Anastrepha ludens* (Diptera: Tephritidae) larvae by *Heterorhabditis bacteriophora* (Rhabditida: Heterorhabditidae) under laboratory and field conditions. Biocontrol Sci. Technol..

[150] Toledo J., Rojas R., Ibarra J.E. (2006). Efficiency of *Heterorhabditis bacteriophora *(Nematoda: Heterrhabditidae) on *Anastrepha serpentina* (Diptera: Tephritidae) larvae under laboratory conditions. Fla. Entomol..

[151] Yee W.L., Lacey A. (2002). Stage-specific mortallity of *Rhagoletis indifferens* (Diptera: Tephritidae) exposed to three species of *Steinernema* nematodes. Biol. Control.

[152] Köppler K., Peters A., Vogt H. (2005). Initial results in the application of entomopathogenic nematodes against the European cherry fruit fly *Rhagoletis cerasi* L. (Diptera: Tephritidae). IOBC/WPRS Bull..

[153] Grunder J. (2006). Final scientific report/COST Action850. Unpublished work, Agrosope ACW, Waedenswil, Switzerland.

[154] Kuske S., Daniel C., Wyss E., Sarraquigne J., Jermini M., Conedera M., Grunder J.  (2005). Biocontrol potential of entomopathogenic nematodes against nut and orchard pests. IOBC/WPRS Bull..

[155] Ovruski S.M., Aluja M., Sivinski J., Wharton R.A. (2000). Hymenopteran parasitoids on fruit infesting Tephritidae (Diptera) in Latin America and the southern United States: diversity, distribution, taxonomic status and their use in biological control. Integr. Pest Manag. Rev..

[156] Hoffmeister T., Aluja M., Liedo P. (1993). The Parasitoid Complexes of Frugivorous Fruit Flies of Central Europe. Fruit Flies: Biology and Management.

[157] Monaco R. (1984). L'*Opius magnus* Fischer (Braconidae), parassita di *Rhagoletis cerasi* L. su *Prunus mahaleb*. Entomologica.

[158] Hadersold O. (1939). Ergebnisse von Parasiten-Zuchten der Zweigstelle Stade der Biologischen Bundesanstalt für Land- und Forstwirtschaft. Arb. Physiol. Angew. Entomol. Berl. Dahlem.

[159] Wiesmann R. (1936). Untersuchungen über die Lebensgeschichte und Bekämpfung der Kirschenfliege *Rhagoletis cerasi* Linné-III. Mitteilung Untersuchungen und Versuche aus dem Jahre 1934. Landw. Jahrb. Schweiz.

[160] Wiesmann R. (1933). Ein Parasit der Kirschfliege (*Rhagoletis cerasi* L.). Mitt Schweiz. Entomol. Ges..

[161] Ahmad M., Carl K. (1966). The natural enemies of *Rhagoletis* in Europe.

[162] Hoffmeister T. (1992). Factors determining the structure and diversity of parasitoid complexes in Tephritid Fruit Flies. Oecologia.

[163] Vogel W. (1950). Untersuchungen über parasitische Hymenopteren der Kirschenfliege. Mitt Schweiz. Entomol. Ges..

[164] Hadersold O. (1938). Ergebnisse von Parasiten-Zuchten der Zweigstelle Stade der Biologischen Reichsanstalt für Land- und Forstwirtschaft. I. Teil: Ichneumonidae. Arb. Physiol. Angew. Entomol. Berl. Dahlem.

[165] Carl K.P. (1968). Collection of and observation on the natural enemies of *Rhagoletis cerasi*.

[166] Sachtleben H. (1934). Deutsche Parasiten der Kirschfruchtfliege (Hym. Ichneumonoidea und Proctotrypoidea). Arb. Morphol. Taxonomische Entomol. Berl. Dahlem.

[167] Wiesmann R. (1935). Die Fauna des Grasbodens unter den Kirschenbäumen. Schweiz. Z. Obst-und Weinbau.

[168] Sajo K. (1902). Die Kirschfliege *Spilographa cerasi*. Pomol. Monatsh..

[169] Schwope D. (1957). Untersuchungen zur Anwendung des Wirkstoffnebelverfahrens bei der Bekämpfung der Kirschfruchtfliege (*Rhagoletis cerasi* L.). Wiss. Z. Martin Luther Univ. Halle Wittenberg.

[170] Boller E., Haisch A., Russ K., Vallo V. (1970). Economic importance of *Rhagoletis cerasi* L., the feasibility of genetic control and resulting research problems. Entomophaga.

[171] Stellwaag F. (1933). Bekämpfung der Kirschfruchtfliege. Anz. Schadl. J. Pest Sci..

[172] Wiesmann R. (1937). Das Wirtspflanzenproblem der Kirschenfliege *Rhagoletis cerasi* L. Landw. Jahrb. Schweiz..

[173] Boller E., Katsoyannos B.I., Hippe C. (1998). Host races of *Rhagoletis cerasi* L. (Dipt., Tephritidae): Effect of prior adult experience on oviposition site preference. J. Appl. Entomol..

[174] Frank B. (1891). Über die Kirschenfliege *Spilographa cerasi* und ihre Bekämpfung. Z. Pflanzenk Pflanz..

[175] Sprengel L. (1931). Die Kirschfliege und ihre wirtschaftliche Bedeutung. Der Obst-und Gemüsebau.

[176] Roessler Y., Robinson A.S., Hooper G. (1989). Insecticidal Bait and Cover Sprays. Insecticidal Bait and Cover Sprays.

[177] Fenili G.A., Zocchi R. (1954). Esperienze di lotta contro la *Rhagoletis cerasi* L. Redia.

[178] Vogel W. (1953). Die Bekämpfung der Kirschenfliege im Jahr 1953. Schweiz. Z. Obst-und Weinbau.

[179] Fenili G.A. (1951). Nuovo contributo alla conoszenza delle biologia della mosca delle ciliege (*Rhagoletis cerasi* L.) ed ai mezzi di lotta cotro di essa. Redia.

[180] Bartolini P., Zocchi R. (1957). Esperienze di lotta contro la *Rhagoletis cerasi* L. nel 1957. Redia.

[181] Galli P. (2004). Die Kirschfruchtfliegenbekämpfung steht auf schmaler Basis. Besseres Obst.

[182] Boller E., Cavalloro R. (1983). Biotechnical Methods for the Management of Fruit Fly Populations. Fruit Flies of Economic Importance.

[183] Agee H.R., Boller E., Remund U., Davis J.C., Chambers D.L. (1982). Spectral sensitivities and visual attractant studies on the Mediterranean Fruit Fly, *Ceratitis capitata* (Wiedemann), Olive Fly, *Dacus oleae *(Gmelin), and the European Cherry Fruit Fly, *Rhagoletis cerasi* (L) (Diptera, Tephritidae). J. Appl. Entomol..

[184] Prokopy R.J., Boller E. (1971). Response of European Cherry Fruit Flies to colored rectangles. J. Econ. Entomol..

[185] Remund U., Boller E. (1978). Kirschenfliegenfallen für Prognosewesen und biotechnische Bekämpfung im Vormarsch. Schweiz. Z. Obst-und Weinbau.

[186] Remund U., Boller E., Cavalloro R. (1983). Pièges Visuels Pour la Lutte Biotechnique et Prévision Négative de la Mouche de la Cerise, *Rhagoletis cerasi* L. Fruit Flies of Economic Importance.

[187] Katsoyannos B.I., Boller E.F. (1980). Second field application of oviposition-deterring pheromone of the European cherry fruit fly, *Rhagoletis cerasi* L. (Diptera, Tephritidae). Z. Angew. Entomol..

[188] Katsoyannos B.I., Boller E.F. (1976). First field application of oviposition-deterring marking pheromone of European cherry fruit fly *Rhagoletis cerasi* L. Environ. Entomol..

[189] Boller E. (1970). Heutiger Stand der Kirschenfliegenforschung in der Schweiz. Schweiz. landw. Forsch..

[190] Blümel S., Russ K., Robinson A.S., Hooper G. (1989). Sterile Insect Technique**-**Manipulation of Races. Fruit Flies Their Biology, Natural Enemies and Control.

[191] Ranner H. (1990). Untersuchungen zur Biologie und Bekämpfung der Kirschfruchtfliege, *Rhagoletis cerasi* L. (Diptera, Trypetidae)-V. Versuche zur Bekämpfung der Kirschfruchtfliege mit Hilfe der Incompatible Insect Technique (IIT). Pflanzenschutzberichte.

[192] Boller E., Ramser E. (1971). Die Zucht der Kirschenfliege (*Rhagoletis cerasi* L.) auf künstlichen Substraten. Schweiz. Z. Obst-und Weinbau.

[193] Katsoyannos B.I., Boller E., Remund U. (1977). Beitrag zur Entwicklung von Methoden für die Massenzucht der Kirschenfliege *Rhagoletis cerasi* L., auf künstlichen Substraten. Mitt. Schweiz. Entomol. Ges..

[194] Köppler K., Kaffer T., Vogt H. (2009). Substantial progress made in the rearing of the European cherry fruit fly, *Rhagoletis cerasi*. Entomol. Exp. Appl..

[195] Köppler K., Peters A., Vogt H. (2003). Erste Ergebnisse zum Einsatz entomopathogener Nematoden gegen die Kirschfruchtfliege *Rhagoletis cerasi* L. DGaaE-Nachrichten.

[196] Köppler K., Storch V., Vogt H., Boos M. (2006). Bait sprays-Eine Alternative zur Bekämpfung der Europäischen Kirschfruchtfliege *Rhagoletis cerasi*?. Proceedings of the 12th International Conference on Cultvation Technique and Phytopathological Probelms in Organic Fruit-Growing.

[197] Luke B.M., Bateman R.P. (2006). Effects of chemical and botanical insecticides used for locust control on *Metarhizium anisopliae var. acridum *conidia after short- to medium-term storage at 30 °C. Biocontrol Sci. Technol..

[198] Depieri R.A., Martinez S.S., Menezes A.O. (2005). Compatibility of the fungus *Beauveria bassiana* (Bals.) Vuill. (Deuteromycetes) with extracts of neem seeds and leaves and the emulsible oil. Neotrop. Entomol..

[199] Tamai M.A., Alves S.B., Lopes R.B., Faion M., Padulla L.F.L. (2002). Toxicidade de produtos fitossanitarios para *Beauveria bassiana *(Bals.)Vuill. Arq. Inst. Biol..

[200] Cavalcanti R.S., Junior A.M., Souza G.C., Arnosti A. (2002). Efeito dos produtos fitossanitarios fenpropatrina, imidiaclopride, Iprodione e tiometoxam sobre o desenvolvimento. Arq. Inst. Biol..

[201] Jaros-Su J., Groden E., Zhang J. (1999). Effects of selected fungicides and the timing of fungicide application on *Beauveria bassiana* induced mortality of the Colorado Potato Beetle (Coleoptera: Chrysomelidae). Biol. Control.

[202] Häseli A., Weibel F., Tamm L. (2005). Überdachungen helfen erfolgreich gegen Pilzkrankheiten. Ökologie&Landbau.

[203] Balmer M. (2005). Kulturschutznetze zur Kontrolle der Kirschfruchtfliege im überdachten Anbau. Obstbau.

[204] Geipel K. (2002). Bekämpfung der Kirschfruchtfliege *Rhagoletis cerasi* L-Abschlussbericht. Bayerische Landesanstalt für Bodenkultur und Pflanzenbau.

[205] FAO FAOSTAT. http://faostat.fao.org/site/567/default.aspx#ancor/.

[206] Arbokost. http://www.acw.admin.ch/themen/00516/01839/01849/index.html?lang=de/.

[207] Müller W. (1970). Agrarmeterologische Untersuchungen über das Erstauftreten der Kirschenfliege (*Rhagoletis cerasi* L.) in Österreich. Pflanzenschutzberichte.

[208] Boller E.F., Remund U. (1983). Dix anées d'utilisation de données sur les sommes de températures journalières pour la prévision des vois de *Rhagoletis cerasi* et d' *Eupoecilia ambiguella* au nord de la Suisse. EPPO Bull..

[209] Russ K., Boller E., Vallo V., Haisch A., Sezer S. (1973). Developement and application of visual traps for monitoring and control of populations of *Rhagoletis cerasi* L. Entomophaga.

[210] Fimiani P., Robinson A.S., Hooper G. (1989). Pest Status-Mediterranean Region. Fruit Flies Their Biology,Natural Enemies and Control.

[211] Schneider F. (1947). Methoden zur Ermittlung des Kirschenfliegenbefalls. Schweiz. Z. Obst-und Weinbau.

[212] Kounatidis I. (2008). Genetic and cytogenetic analysis of the fruit fly *Rhagoletis cerasi* (Diptera: Tephritidae). Genome.

[213] Arthofer W., Riegler M., Schneider D., Krammer M., Miller W.J., Stauffer C. (2009). Hidden Wolbachia diversity in field populations of the European cherry fruit fly, *Rhagoletis cerasi* (Diptera, Tephritidae). Mol. Ecol..

[214] Levinson H.Z., Haisch A., Cavalloro R. (1983). Optical and Chemosensory Stimuli Involved in Host Recognition and Oviposition of the Cherry Fruit Fly *Rhagoletis cerasi* L. Optical and Chemosensory Stimuli Involved in Host Recognition and Oviposition of the Cherry Fruit Fly *Rhagoletis cerasi* L.

[215] Cirio U., Vita G. Natural chemical substances affecting Cherry Fruit Fly behavior. I. Laboratory trials. Proceedings of the 5th meeting of the IOBC working group on *Rhagoletis cerasi*.

